# Biomaterials used for tissue engineering of barrier-forming cell monolayers in the eye

**DOI:** 10.3389/fbioe.2023.1269385

**Published:** 2023-09-27

**Authors:** Samantha Sasseville, Samira Karami, Ange Tchatchouang, Pascale Charpentier, Princia Anney, Delphine Gobert, Stéphanie Proulx

**Affiliations:** ^1^ Axe Médecine Régénératrice, Hôpital du Saint-Sacrement, Centre de Recherche en Organogénèse Expérimentale de l’Université Laval/LOEX; Centre de Recherche du Centre Hospitalier Universitaire (CHU) de Québec-Université Laval, Québec, QC, Canada; ^2^ Département d’ophtalmologie et d’oto-rhino-laryngologie-chirurgie cervico-faciale, Faculté de Médecine, Université Laval, Québec, QC, Canada; ^3^ Centre universitaire d’ophtalmologie (CUO), Hôpital du Saint-Sacrement, CHU de Québec-Université Laval, Québec, QC, Canada

**Keywords:** corneal endothelium, retinal pigment epithelium (RPE), tissue engineering, biomaterials, scaffolds

## Abstract

Cell monolayers that form a barrier between two structures play an important role for the maintenance of tissue functionality. In the anterior portion of the eye, the corneal endothelium forms a barrier that controls fluid exchange between the aqueous humor of the anterior chamber and the corneal stroma. This monolayer is central in the pathogenesis of Fuchs endothelial corneal dystrophy (FECD). FECD is a common corneal disease, in which corneal endothelial cells deposit extracellular matrix that increases the thickness of its basal membrane (Descemet’s membrane), and forms excrescences (guttae). With time, there is a decrease in endothelial cell density that generates vision loss. Transplantation of a monolayer of healthy corneal endothelial cells on a Descemet membrane substitute could become an interesting alternative for the treatment of this pathology. In the back of the eye, the retinal pigment epithelium (RPE) forms the blood-retinal barrier, controlling fluid exchange between the choriocapillaris and the photoreceptors of the outer retina. In the retinal disease dry age-related macular degeneration (dry AMD), deposits (drusen) form between the RPE and its basal membrane (Bruch’s membrane). These deposits hinder fluid exchange, resulting in progressive RPE cell death, which in turn generates photoreceptor cell death, and vision loss. Transplantation of a RPE monolayer on a Bruch’s membrane/choroidal stromal substitute to replace the RPE before photoreceptor cell death could become a treatment alternative for this eye disease. This review will present the different biomaterials that are proposed for the engineering of a monolayer of corneal endothelium for the treatment of FECD, and a RPE monolayer for the treatment of dry AMD.

## 1 Introduction

**FIGURE 1:**
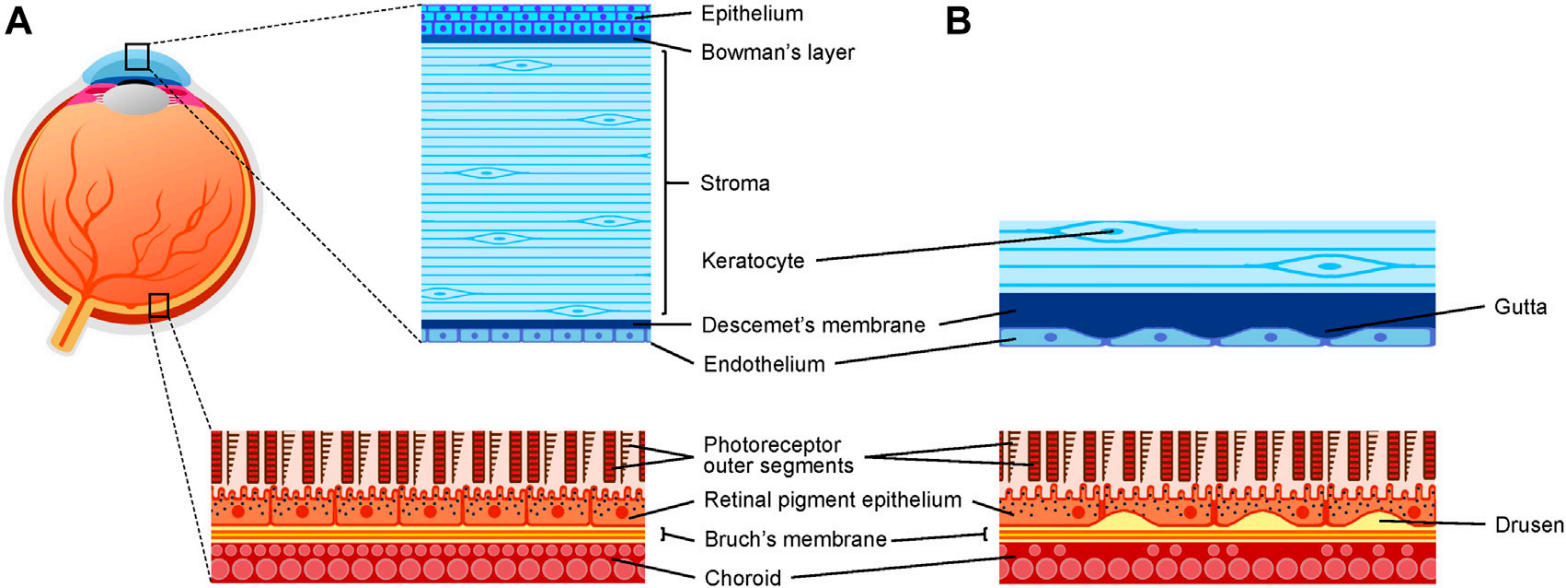
Schematic representation of the eye and two barrier-forming tissues in health and disease. **(A)** Schematic representation of the cornea and the RPE/choroid in healthy conditions. **(B)** Schematic representation of the posterior cornea in Fuchs endothelial corneal dystrophy and of the RPE/choroid in AMD.

In the anterior portion of the eye, the corneal endothelium forms a barrier between the aqueous humor of the anterior chamber and the corneal stroma. In the posterior portion of the eye, the retinal pigment epithelium (RPE) forms a barrier between the choroidal blood supply and the photoreceptors. Both are made out of highly metabolically active cells that form a tightly packed cell monolayer. Both are also prone to age-related degenerative diseases, such as Fuchs endothelial corneal dystrophy (FECD) and dry age-related macular degeneration (dry AMD), in which extracellular matrix deposits (called guttae in the case of FECD, and drusen in the case of dry AMD) form underneath the monolayer, affecting their barrier integrity (Figure 1). In both cases, cell transplantation of healthy cells could become a treatment alternative. Transplantation of cells in suspension is feasible, however, once injected into the eye, they have to quickly adhere, survive, and reform a functional monolayer, which can be a challenge, especially in the context of a diseased environment. Therefore, an alternative would be to transplant an already functional cell monolayer. However, the fragile nature of these monolayers requires that they be transplanted using a biomaterial that will provide support.

On top of the common properties that biomaterials must have in terms of safety and biocompatibility, biomaterials intended for eye tissue replacement must take into account a curved shape, as both the cornea and the retina are curved structures. To fit perfectly in the eye, the biomaterial must also have the right size and thickness to limit folds, prevent graft displacement, and obtain optimal visual outcome. Abnormal curvature and folds can lead to partial or total graft detachment and loss of visual acuity. For barrier-forming cells, such as the corneal endothelium or the RPE, cells need to mature into a permeable barrier to support their physiological function. Depending on the biomaterial, this can include an additional step of surface modification to incorporate bioactive molecules or specific chemical cues to enhance cell-substrate interactions. The selected biomaterial also needs to consider the controlled permeability and transport properties to mimic the selective barrier function of the native monolayer. Finally, in the case of the corneal endothelium, the biomaterial also needs to be transparent, and should not support neovascularisation as it could lower visual acuity, create inflammation, diminish immune privilege, and lead to graft failure and rejection (Bachmann et al., 2010).

Cells can perceive the mechanical proprieties of their substrate and respond to it. Therefore, the biomaterial should have mechanical properties that mimic the native environment, providing appropriate stiffness and elasticity to support cell function. Mimicking the mechanical cues of the native environment can aid in the polarization and functional maturation of both corneal endothelial (Palchesko et al., 2015; Du et al., 2022) and RPE cells (Engler et al., 2006; Wen et al., 2014; Smith et al., 2018).

## 2 The corneal endothelium and Descemet’s membrane

The cornea is the transparent tissue in the front part of the eye. From the outside-in, it is composed of a pluristratified epithelium, Bowman’s layer, a thick stroma (with resident cells called keratocytes), Descemet’s membrane, and an endothelium (Figure 1). Descemet’s membrane is the basal membrane of the corneal endothelium, and therefore is the optimal scaffold to mimic for the engineering of this monolayer. Its properties are described in Table 1. The properties of Descemet’s membrane send cues to the CECs and may in turn affect the corneal endothelium’s barrier integrity and overall functionality. The main function of the corneal endothelium is to maintain the corneal stroma in a partially dehydrated state, called deturgescence, which is important for stromal transparency. Corneal endothelial cells form a leaky barrier, allowing nutrients from the aqueous humor to reach the posterior keratocytes. The leaky barrier is attributed, at least in part, by the presence of the discontinued tight junction protein ZO-1 around the cells (Petroll et al., 1999). Intercellular junctions also include gap and adherens junctions (Petroll et al., 1999). To counterbalance the influx of liquid into the stroma, endothelial cells actively transport fluid back to the anterior chamber, which is achieved through Na^+^K^+^-ATPase pumps and other ion transporters (Bonanno, 2012). Corneal transparency thus depends on the endothelium’s ability to maintain a pump/leak balance. This role is also dependent on cell density. *In vivo*, CECs are arrested in the G1 phase of the cell cycle (Joyce et al., 1996). Since they do not proliferate *in vivo*, age-related changes lead to a decrease in cell density (Laing et al., 1976). In older adults, the cell density averages 2,500 cells/mm^2^ (Yee et al., 1985; Bourne et al., 1997). Following surgery or in a diseased state, cell density can fall below a critical threshold (400–500 cells/mm^2^) (Price et al., 2021) and excess fluid can enter the stroma, leading to corneal edema and disorganization of stromal collagen fibers, which becomes less transparent, resulting in vision loss. This is known as bullous keratopathy.

**TABLE 1:** Characteristics of the optimal biomaterial for the engineering of the corneal endothelium and the retinal pigment epithelium.

Characteristics	Corneal endothelium (Descemet’s membrane substitute)	Retinal pigment epithelium (Bruch’s membrane substitute)	References
THICKNESS	5 µm (age 10)	2–4 µm	Johnson et al. (1982), Fields et al. (2020), Hammadi et al. (2023)
	13 µm (older adult)		
ECM FACING THE CELLS	Collagen IV & XII	Chondroitin sulphate, Heparan sulphate	Kabosova et al. (2007), Murphy et al. (2020)
	Laminin 511	Collagen IV	
	Nidogen-1, Thrombospondin-1, Perlecan	Laminin	
		Fibronectin	
PERMEABILITY	Permeable to large macromolecules	Semi-permeable to biomolecules, nutrients, water, oxygen, and metabolic waste	Kim et al. (1971), Booij et al. (2010)
ELASTICITY	20–80 kPa (mean 50 ± 17,8 kPa)	7–19 MPa	Last et al. (2009), Hammadi et al. (2023)

TOPOGRAPHY	Uniform topography	Presence of native basal lamina on the apical surface of the membrane	Del Priore and Tezel, (1998); Last et al., (2009)
	Densely packed basement membrane, with pores of 38 nm diameter		
TRANSPARENCY	Important	Not important	Bachmann et al. (2010)
SHAPE	Curved shape	Curved shape	Kimoto et al., (2014); Murphy et al., (2020)
NON-IMMUNOGENICITY	Important	Important	Lee et al., (2016); Rohiwal et al., (2021)
ADHESIVENESS	Important	Important	Tezel and Del Priore, (1997); Parekh et al., (2021)

### 2.1 Bullous keratopathy

Bullous keratopathy is characterized by corneal endothelial decompensation associated with irreversible stromal edema. Conditions or events that give rise to bullous keratopathy include pseudophakic bullous keratopathy (PBK), a sterile or infectious inflammation, or FECD (Morishige and Sonoda, 2013). Endothelial decompensation in PBK is thought to be multi-factorial and can be caused by intra-operative surgical trauma or post-operative events (inflammation, high intra-ocular pressure) following intra-ocular surgery. Contrary to PBK (where the endothelium is not diseased), FECD manifests by the progressive thickening of Descemet’s membrane, and the development of extracellular matrix-related excrescences, called guttae, on its endothelial surface, that can be embedded by a fibrillar layer at later stages (Figure 1). Concurrently, there is a decrease in cell density. Due to this cell loss, the corneal endothelium loses its integrity and is not able to maintain stromal deturgescence, which will create stromal edema and painful epithelial bulla (Elhalis et al., 2010). Evidence that FECD Descemet’s membrane constitutes a toxic environment for the cells are numerous (Rizwan et al., 2016; Kocaba et al., 2018; Hribek et al., 2021), which supports the importance of removing the diseased Descemet’s membrane, and thus also increases the need for the development of a Descemet’s membrane substitute.

### 2.2 Current treatments and limits

PBK and FECD are the first common causes of corneal transplantation worldwide (Ghosheh et al., 2008; Gain et al., 2016; Matthaei et al., 2017; Iselin et al., 2021). The current treatment is to replace the dysfunctional corneal endothelium with a healthy one through corneal transplantation. Descemet’s stripping automated endothelial keratoplasty (DSAEK) and Descemet’s membrane endothelial keratoplasty (DMEK) are currently the two preferred surgical techniques for corneal endothelial transplantation, with both techniques presenting fewer risks of surgical complications and permitting faster visual recovery with a lower rate of graft rejection than full thickness grafting techniques such as penetrating keratoplasty (Turnbull et al., 2016).

Despite the high success rate of DSAEK/DMEK, important issues remain, such as graft dislocation (Price and Price, 2006; Trindade and Eliazar, 2019), immunological rejection (Price et al., 2009; Hos et al., 2017; Price et al., 2018) and primary donor failure (Deng et al., 2018), which may require surgical revisions, prolonged corticosteroid treatment or re-grafting. DMEK/DSAEK surgical procedures are invasive, and their long-term clinical results remain unclear. Furthermore, corneal transplantation and eye banking facilities are not equally available around the world and most countries suffer from a severe shortfall of donated tissues. Globally, 12.7 million patients await transplantation, with only 1/70 grafted (Gain et al., 2016). The limited quantity of high-quality donor tissue and their high demand are limiting factors justifying the need for the development of alternative therapies. A promising approach is to expand donor CECs *in vitro* (allowing cells from one donor cornea to treat many patients), seed them on a compatible biomaterial, and transplant them as a functional endothelial monolayer.

## 3 Tissue engineering of the corneal endothelium

Over the years, different types of biomaterials have been proposed for the engineering of the corneal endothelium. They include biomimetic substrates (man-made materials that provide an architectural framework reminiscent of native extracellular matrix), natural membranes that have been repurposed for the engineering of the corneal endothelium, and synthetic scaffolds that are typically made of polymeric biocompatible materials. Engineering of the corneal endothelium also requires high quality cells that will reform a functional monolayer.

### 3.1 Cell source

As a proof of concept, immortalized CECs (Bednarz et al., 2000; Schmedt et al., 2012) can be used to verify that cells adhere and reform a monolayer on the tested biomaterial. However, moving towards human implantation, untransformed primary cells should be privileged, as immortalized CECs have been modified to increase their proliferative potential.

Proliferation of human CECs can be induced *in vitro* by disrupting contact inhibition and adding growth factors (Joyce and Zhu, 2004). Unfortunately, this expansion is limited, and cells tend to lose their endothelial phenotype following repeated passaging (Roy et al., 2015), a process attributed to endothelial-to-mesenchymal transition (Engelmann et al., 1988; Engelmann et al., 2004; Zhu and Joyce, 2004; Okumura et al., 2013; Roy et al., 2015). This change in phenotype upon culture can be explained by the partial mesenchymal nature of CECs. Indeed, native corneal endothelial cells express mesenchymal markers, such as vimentin (Foets et al., 1990; He et al., 2016) and n-cadherin (Zhu et al., 2008). On top of mesenchymal markers, patches of cells in the corneal endothelium also display epithelial markers, such as keratins 8 and 18 (Foets et al., 1990; Merjava et al., 2009; He et al., 2016), and neuronal markers, such as neuron specific enolase (NSE) (Hayashi et al., 1986). The heterogenous nature of the corneal endothelium was recently been brought to light using scRNAseq analysis, where 2 to 4 distinct subpopulations were identified in the native tissue (Català et al., 2021; Wang et al., 2022), and up to seven in primary cultures of CECs, including some clusters with senescent or fibrotic phenotypes (Català et al., 2023). CECs with a fibroblastic phenotype will not be able to form a homogenous monolayer capable of forming the cellular junctions essential for the functionality of the endothelium (Roy et al., 2015). Many studies have thus addressed the influence of cell culture conditions in order to maintain an endothelial phenotype, including different cell isolation techniques (Li et al., 2007; Choi et al., 2014; Santerre and Proulx, 2022), cell culture media formulation (reviewed in (Peh et al., 2011)), and dual media approaches (Peh et al., 2015; Beaulieu Leclerc et al., 2018). Maintaining an endothelial phenotype is key in regenerative medicine for preserving the cell monolayer’s physiological function. Cell morphology is a good indicator of an endothelial phenotype. It can be measured by calculating cell circularity based on the area and the perimeter of the cells (Circularity index = 4π × Area/Perimeter^2^) (Peh et al., 2013; Ang et al., 2016). Using this formula, a round cell will have a circularity index of 1, a hexagonal cell will have a circularity index of 0,88, and a fibroblastic cell will have a circularity index below 0,55 (Xu et al., 2021).

However, cell morphology is not sufficient to determine cell quality. Using immunofluorescence and flow cytometry, mature CECs can be distinguished according to their expression of specific cell markers. Cells can be evaluated by their expression profile of specific clusters of differentiation (CD). Mature differentiated CECs should express the following profile of CD; CD166^+^, CD44^−/dull^, CD24^−^, and CD105^−^ (Kitazawa et al., 2023). Some membrane proteins also can be used as markers as they are only expressed in mature CECs, such as SLC4A11, which is only expressed in a low mitogenic environment and low passage *in vitro* (Frausto et al., 2020).

The limited expansion of primary cells can also be circumvented using stem cells. Many types of stem cells can be differentiated into CEC-like cells. Unipotent stem cells can be isolated by sphere-forming assay (Yokoo et al., 2005; Amano et al., 2006; Mimura et al., 2010). However, these progenitor cells may acquire epigenetic modifications through passages leading to inhibition of proliferation and differentiation (Navaratnam et al., 2015). Pluripotent embryonic stem cells (ESCs) (Hanson et al., 2017) and induced pluripotent stem cells (iPSCs) (Wagoner et al., 2018) can also be used but are associated with ethical concerns (King and Perrin, 2014) and teratoma formation (Lee et al., 2009). Alternatively, CEC-like cells can be differentiated from adult multipotent mesenchymal stem cells (MSCs) isolated from umbilical cord blood (Joyce et al., 2012), bone marrow (Shao et al., 2011), corneal stroma (Ju et al., 2012; Hatou et al., 2013), and skin (Inagaki et al., 2017; Shen et al., 2017; Gutermuth et al., 2019), therefore negating the ethical concerns and teratoma formation risen by pluripotent cells. The use of stem cells eliminates the need for donor ocular tissue. It opens the door for autologous treatment, but further research is needed to characterize the precursor cells and their derivates. Furthermore, genetic editing techniques such as CRISPR could allow autologous treatment of patients with genetic endotheliopathies, such as FECD.

### 3.2 Biomimetic substrates

Collagen is an abundant protein in most extracellular matrices (Ricard-Blum, 2011), and Descemet’s membrane is no different (de Oliveira and Wilson, 2020). Collagen and its denatured form, gelatin, are widely used in the tissue engineering of many organs because of their biocompatibility, biodegradability, hydrophilicity, and affordability (Rezvani Ghomi et al., 2021). However, they tend to be fragile and swell when maintained in aqueous conditions, leading to decreased transparency (Watanabe et al., 2011). Cross-linking increases chemical bonds between the collagen fibrils, therefore improving mechanical strength (Jumblatt et al., 1980; McCulley et al., 1980; Mimura et al., 2004b; Kimoto et al., 2014; Rizwan et al., 2017). Unfortunately, many cross-linking techniques require chemical compounds that may cause toxicity to the cultivated cells or the host. Alternatively, collagen can be compressed, making the biomaterial sturdier without chemical cross-linking (Levis et al., 2012; Cen and Feng, 2018). Another derivate of collagen used in corneal endothelium bioengineering is Vitrigel (Koizumi et al., 2008; Yoshida et al., 2014; Yoshida et al., 2017). The latter is produced by gelation, vitrification, and rehydration of collagen type I, creating a resistant thin transparent membrane (Takezawa et al., 2004).

Since collagen is a large protein, that is, hard to manipulate, collagen-like peptides (CLPs) are being studied as short peptides able to self-assemble into triple helices mimicking full-length collagen hydrogels (Wang et al., 2008; Stahl et al., 2010; O'Leary et al., 2011; Luo and Kiick, 2013). CLPs combined with polyethylene glycol (CLP-PEG) have already been grafted into mini-pig models as corneal stromal replacement and demonstrated good biocompatibility and sufficient mechanical strength (Jangamreddy et al., 2018). Pilot studies have demonstrated that this biomaterial allows adhesion and proliferation of CECs (Sasseville et al., 2022). This material showed excellent potential for future graft but still requires optimization as long-term culture was difficult. An advantage of this approach is that short extracellular matrix (ECM) sequences can be added in the hydrogel. For example, the laminin-derived peptide sequence YIGSR can be cross-linked into the hydrogel, which could help CECs to remain onto the biomaterial.

Silk fibroin membranes have also been extensively used for their biodegradability, non-immunogenicity, and inherent optical and mechanical attributes (Cao and Wang, 2009; Lee et al., 2016). *In vitro*, silk membranes can support human (Madden et al., 2011; Ramachandran et al., 2020) and rabbit (Kim et al., 2018; Song et al., 2019) CEC maturation. Silk has also been used in an *in vivo* rabbit model and could recover corneal transparency over a 6-week follow-up period (Vazquez et al., 2017). Furthermore, Hazra et al. demonstrated that despite the much greater tensile strength of silk membrane compared to native Descemet’s membrane, human CECs secreted similar ECM proteins and exhibited endothelial phenotype in long-term cell culture (Hazra et al., 2023). Long-term *in vivo* studies are still needed to evaluate safety and performance.

Chitosan is a polysaccharide derived from crustaceans. It is biocompatible, biodegradable, antibacterial, antifungal, and easy to fabricate, but it is fragile and hydrophobic. For those reasons, chitosan is often used combined with other compounds. Polycaprolactone (PCL) is a polymer that can be added to chitosan without extensive chemical modifications (Sarasam and Madihally, 2005) and allows CEC culture (Wang et al., 2012; Young et al., 2014; Wang et al., 2019). However, chitosan-PCL scaffolds can be fragile. Supplementing the model with chitosan nanoparticles can amplify its mechanical strength (Tayebi et al., 2021).

A mature monolayer of CECs has also been achieved using a self-assembled corneal stromal substitute (Proulx et al., 2010; Jay et al., 2015; Bourget and Proulx, 2016). The biocompatibility of the corneal stromal substitute was assessed by intrastromal transplantation in a cat model and showed no sign of rejection, inflammation, and toxicity over a four-month follow-up period (Boulze Pankert et al., 2014). The self-assembly approach is based on the inherent capacity of fibroblasts to secrete and assemble their own ECM when cultured in the presence of serum and ascorbic acid. This technique has been used to produce tissue-engineered bone, skin, connective and adipose tissues, cornea, and choroidal substitutes (Proulx et al., 2010; Labbé et al., 2011; Boa et al., 2013; Galbraith et al., 2017; Djigo et al., 2019). It is a great alternative that does not require exogenous biomaterials and that could be produced using the patient’s own cells, which would avoid graft rejection.

### 3.3 Natural membranes

In an effort to be as biocompatible as possible, native thin membranes have also been proposed as biomaterials for the engineering of the corneal endothelium. Unlike biomimetic substrates, they require organ donation but have a more complex composition that can better mimic the Descemet’s membrane composition. However, their allogenic/xenogeneic nature and their mechanical resistance can be issues.

Denuded amniotic membranes have been used as a corneal endothelium biomaterial. While the transparency is sub-optimal, endothelialized amniotic membranes have been grafted in rabbits (Ishino et al., 2004) and cats (Fan et al., 2013), leading to decreased corneal edema and increased transparency as opposed to ungrafted eyes. Recently, Zhao et al. (2022) explored cross-linking as a way of enhancing the mechanical strength of amniotic membranes and ECM protein coating to improve cell culture. They found increased mechanical strength with cross-linking, improved adhesion, and proliferation with ECM coating, and return of transparency when grafted into cats and monkeys (Zhao et al., 2022).

The anterior lens capsule is a transparent thick basement membrane with a similar collagen composition (type IV collagen and laminin α5 and β2) as the posterior surface of the Descemet’s membrane (Nakayasu et al., 1986; Marshall et al., 1991; Ronci et al., 2011) and is compatible with integrin binding of CECs (Choi et al., 2013). Human (Yoeruek et al., 2009) and pig (Spinozzi et al., 2019) CECs can reform an endothelial monolayer and can sustain corneal transparency when grafted into pigs (Spinozzi et al., 2019). The main downfall with anterior lens capsule as a biomaterial is donor dependant variability.

Tilapia is a fish that possesses scales composed of type I collagen (Chen et al., 2011). Once decalcified and decellularized, fish scales form a biodegradable (van Essen et al., 2013), transparent (Ikoma et al., 2003) material that can be cellularized with or without ECM protein coating (Parekh et al., 2018; Hsueh et al., 2019; Li et al., 2020).

Descemet’s membrane itself can be used as a carrier. Indeed, Descemet’s membrane was the first carrier used for the engineering of the corneal endothelium (Jumblatt et al., 1978; Gospodarowicz et al., 1979). At the time, corneal buttons were excised and deendothelialized using a cotton swab. The cornea with the remaining denuded Descemet’s membrane was seeded with cells, maintained in culture, and grafted through full thickness keratoplasty. Since then, improvements have been made and the reconstructed endothelium is grafted using only Descemet’s membrane (Lu et al., 2020). Nonetheless, human Descemet’s membranes are limited due to donor shortage and animal Descemet’s membranes represent a risk of xenograft rejection.

### 3.4 Synthetic scaffolds

Biologic and biomimetic biomaterials can have high variation between batches. Synthetic substrates could therefore be a great alternative for low variability and highly pure and defined materials. They can also be great off-the-shelf options that are mechanically strong and have tunable degradation time and porosity. Many have been tested; Poly (methyl-methacrylate) (PMMA), polycaprolactone (PCL), poly (lactic-co-glycolic acid) (PLGA) (Kruse et al., 2018), poly-ε-lysine (Kennedy et al., 2019), and poly (ethylene glycol) (PEG) (Ozcelik et al., 2014) and have been shown to allow endothelial monolayer formation *in vitro*. However, many synthetic polymers are hydrophobic. This can be an issue since hydrophobic substrates tend to have low permeability to small hydrophilic molecules such as glucose, little to no degradation in aqueous conditions and low biocompatibility (Kruse et al., 2018). Biomaterials with low degradation can raise long-term toxicity concerns. For example, PMMA is non-degradable and shows long-term cytotoxicity (Kruse et al., 2018). Albeit, PCL has a slow degradation rate but breaks down in small non-toxic molecules that can be evacuated form the aqueous humor, causing no toxicity (Sun et al., 2006). To improve biocompatibility, synthetic scaffolds can be crosslinked to ECM proteins or peptides to enhance chemical interactions between the cell membrane and the substrate (Palchesko et al., 2015; Kennedy et al., 2019). They can also be amalgamed with biomimetic compounds to improve overall mechanical strength and biocompatibility (Young et al., 2014; Chen et al., 2015; Van Hoorick et al., 2020; Himmler et al., 2021; Tayebi et al., 2021). A way to incorporate synthetic materials to biomimetic compounds is by layering. Van Hoorick et al. used a thin layer of poly (D,L-lactic acid) (PDLLA), a FDA-approved biodegradable polymer, to mechanically enhance gelatin sheets (Van Hoorick et al., 2020). They were able to obtain a transparent, glucose permeable 1 µm thick membrane with a Young’s modulus comparable to the native Descemet’s membrane. Song et al. (2022) layered poly (lactide-co-caprolactone) (PLCL) on top of decellularized ECM synthetized by umbilical cord blood mesenchymal stem cells. They were able to obtain a robust transparent 20 µm thick membrane. Both layered synthetic-biological membranes were able to sustain immortalized and primary human CEC culture exhibiting proper morphology and function-related proteins. Nonetheless, further studies are still necessary to ensure long term safety and efficiency.

### 3.5 Functionality assessments of the engineered corneal endothelial monolayer

#### 3.5.1 *In vitro* functionality assays

After demonstrating that cells attach and proliferate onto the biomaterial, the next step is to assess the functionality of the reformed monolayer. For the corneal endothelium, these functionality assessments include the expression of function-related markers, the integrity of the barrier, and the ability of the monolayer to pump fluid out of the stroma.

The expression of barrier-related proteins, such as tight junction proteins ZO-1, claudin-10b and adherent junction protein N-Cadherin, and their proper cytolocalization at the cellular membrane, is a good indicator of barrier formation (Petroll et al., 1999; Beebe and Coats, 2000; Zhu et al., 2008; Inagaki et al., 2013). Their expression is essential for the integrity of the corneal endothelium by inhibiting the cell proliferation pathway, resulting in the conservation of a cellular monolayer (Joyce et al., 2002). Their expression is also a good indicator of CEC maturity as they are not expressed at the membrane during the *in vitro* proliferation phase. Other function-related proteins to study include Na + K + -ATPase, SLC4A11 and NBCe1. They are three membrane proteins that support the transport of ions across CECs cell membranes and help to generate a transendothelial osmotic gradient which facilitates the “pump mechanism” of the corneal endothelium (Dikstein and Maurice, 1972; Damkier et al., 2007; Liu et al., 2010; Bonanno, 2012).

While immunofluorescence and flow cytometry can highlight the presence of ionic pumps and cellular junctions, permeability assays can demonstrate their functionality. These tests are inspired by the one used for vascular endothelium (Huynh et al., 2011). Briefly, fluorescent dextran is added to the media, and the permeability is evaluated based on the capacity of the reformed endothelium to let the dextran penetrate the monolayer and the underlying scaffold over time (Beaulieu Leclerc et al., 2018; Theriault et al., 2018). Na^+^K^+^-ATPase functionality can also be evaluated using a bioreactor that mimics intraocular conditions, such as intraocular pressure and flow of aqueous humor (Theriault et al., 2019; Anney et al., 2021). A functional endothelium will lead to stromal deswelling, which will lead to a more transparent cornea, as well as a thinner cornea. Corneal thickness can be measured using histology cross-sections (Theriault et al., 2019). Alternatively, intrafibrillar collagen spacing can me calculated from transmission electron microscopy images (Theriault et al., 2019). Transmission electron microscopy can also be used to document the presence of tight and adherens junctions (Proulx et al., 2009a).

#### 3.5.2 *In vivo* functionality assays (pre-clinical studies)

##### 3.5.2.1 Animal models

The ultimate proof of the functionality of an engineered corneal endothelium is its ability to unswell the stroma (deturgescence) and return/maintain corneal transparency following transplantation. Rabbits are often used as models because of the size of their cornea (radius of 7.26 mm) (Bozkir et al., 1997). They are easily available, relatively easy to manipulate, and have eyes big enough for surgery, unlike rats or mice. However, contrary to humans, rabbit CECs maintain proliferative capabilities *in vivo* (Van Horn et al., 1977). It is, therefore, important to ensure that the restoration of transparency is due to the grafted cells and not the natural reendothelialization of the host (see Section 3.5.2.4).

Compared to rabbits, cats are a good model due to their intrinsic lack of CEC proliferation *in vivo* (Van Horn et al., 1977). However, their Descemet’s membrane is highly adhered to the stroma, making it difficult to perform a graft using DSAEK/DMEK. This explains why researchers that used the feline model transplanted their engineered tissue by means of penetrating keratoplasty (Proulx et al., 2009b; Haydari et al., 2012). Laboratory cats are also less available and are much more expensive to buy and house.

Pigs are often used as a model for their eyes because of their physiological and anatomical resemblance to the human eye. Pigs are also widely available and low-cost but are rarely used for corneal transplantation. Brunette et al. evaluated the pig and cat models for penetrating keratoplasty (Brunette et al., 2011). They found increased inflammation, edema, corneal thickness, and decreased transparency post-operatively in pigs, as opposed to cats. They also noted smoother recovery from general anesthesia in cats.

Monkeys are the closest physiological model to humans as they also do not regenerate their corneal endothelium *in vivo* (Van Horn and Hyndiuk, 1975), but have a higher cell density than humans (Ollivier et al., 2003). Monkeys are also very expensive and intricate to care for, as very few laboratories have the proper infrastructure for their housing.

##### 3.5.2.2 Surgical considerations

While many studies have used the rabbit as a model for the transplantation of CECs, it is important to note that this model does present several differences when compared to the human eye. First, mean central corneal thickness in the adult rabbit is significantly thinner than in humans, making the cornea much more pliable (Chan and Haschke, 1982). Consequently, maintaining the anterior chamber during surgery, as well as ensuring watertightness of the surgical incisions can be more challenging, even for an experienced surgeon. Post-operatively, rabbits are more prone to exhibit an exuberant inflammatory reaction which could hamper the adhesion of tissue-engineered corneal endothelium, and care must be taken during surgery to minimize the formation of fibrin in the anterior chamber (Crouzet et al., 2022).

##### 3.5.2.3 Post-operative follow up

Once grafted, the same instruments used in clinic can be used to evaluate the grafted endothelium in the living animal in a non-invasive way, allowing to document its evolution over time. A slit lamp is an optical instrument that can be used to evaluate the three-dimensional anatomy of the anterior segment of the eye (Yuan et al., 2015; Martin, 2018; Kaur and Gurnani, 2023). It can also be used to assess corneal transparency and thickness, as well as estimate inflammation levels in the anterior chamber, and signs of rejection (Kaur and Gurnani, 2023). The corneal endothelium can also be imaged by specular microscopy. With this instrument, it is possible to photograph the endothelial monolayer, assess cell morphology, and calculate their density (Benetz and Lass, 2018). Optical coherence tomography is another non-invasive instrument that can take a high-resolution cross-sectional picture of the cornea (Venkateswaran et al., 2018). It can be used to assess graft positioning or detachment, endothelial irregularities, and pachymetry. Pachymetry is the measurement of corneal thickness, which reflects stromal hydration. To maintain a transparent cornea, the stroma needs to be in a state of partial deturgescence. Infiltration of liquid into the stroma will lead to stromal edema and loss of transparency. Experimental success is usually defined by decreased corneal thickness to its pre-operative value.

##### 3.5.2.4 Post-mortem analysis

The grafted cornea can be harvested and analyzed to document the corneal endothelium and the biomaterial’s integrity. While classic *in vitro* characterization analysis (histology, immunostaining of function-related proteins, scanning or transmission electron microscopy) can be performed, additional tests might be necessary. When a bioengineered corneal endothelium is grafted into an animal model with known *in vivo* proliferative capabilities (e.g., rabbit), it is crucial to demonstrate that the reformed endothelium is issued from the graft and not from the animal itself. For this particular purpose, grafted cells can be fluorescently labeled (Joo et al., 2000; Mimura et al., 2004a; Hsiue et al., 2006), distinguishing non-fluorescent native cells from grafted fluorescent cells. Karyotyping (Gospodarowicz et al., 1979; Lagali et al., 2010) or specie-specific antibodies can also differentiate grafted cells.

### 3.6 Alternative techniques to tissue engineering of the corneal endothelium

In parallel to tissue engineering of the corneal endothelium, other groups have focused their efforts on reforming a functional corneal endothelium by injecting CECs into the anterior chamber, thereby bypassing the need for a scaffold. While most pre-clinical studies have focused on the injection of cultured CECs (Mimura et al., 2005; Bostan et al., 2016; Okumura et al., 2018), it has also been suggested that injection of noncultured CECs could reform a functional endothelium and restore corneal transparency. Interestingly, this process, described as simple noncultured endothelial cells injection (SNECi), represents a simplified method when compared to tissue engineering or cultured endothelial cell injection techniques, as it does not necessitate the proliferation of cells that need to maintain an endothelial phenotype throughout the cell culture process (Ong et al., 2020). Moreover, it was noted that with both cultured and noncultured cells injection techniques, results were comparable to those of studies using tissue engineered corneal endothelium (Peh et al., 2019). However, it was also noted that to promote adhesion of injected cells to Descemet’s membrane, strict prone positioning had to be maintained for at least 3 h after surgery (Mimura et al., 2007; Peh et al., 2019) and techniques to enhance CEC delivery and adhesion to the inner cornea such as the use of magnetic particles might be necessary (Moysidis et al., 2015; Xia et al., 2019).

On the other hand, although it is generally accepted that both endothelial cell injection and tissue engineered endothelium techniques will help alleviating the global shortage of donor corneas as compared to more traditional techniques such as DSAEK/DMEK, it is unclear yet if either of these techniques will be more cost-effective than the other (Soh et al., 2021; Kaufman and Jun, 2023). Nonetheless, tissue engineering offers some theoretical advantages, such as a more finely tuned targeted delivery to the diseased area. It is also likely that patients with a more advanced disease, for example, patients with advanced FECD in whom the Descemet’s membrane is severely compromised, might benefit more from Descemet’s membrane stripping, guttae removal and replacement of the endothelium using a tissue engineered corneal endothelium grown on a scaffold, rather than using direct intracameral injection of CECs.

## 4 The retinal pigment epithelium and Bruch’s membrane

The retina is the neurosensitive tissue in the back of the eye. It consists of two main parts, the neuroretina and the RPE. The neuroretina is composed of glial cells (Muller cells) and different neuronal cells, namely, ganglion, horizontal, bipolar, amacrine, and photoreceptor cells. Photoreceptors are in direct contact with the RPE. The RPE is a monolayer of regularly arranged cuboidal epithelial cells that are tightly packed owing to tight junctions (ZO-1) between cells. RPE cells along with the underlying Bruch’s membrane form the outer blood-retinal barrier (oBRB) that separates the choroid blood supply from the photoreceptors of the outer retina (Figure 1).

Bruch’s membrane is a thin (2–4 µm), elastic, acellular, fibrous, and semi-permeable extracellular matrix with a pentalaminar structure that separates the RPE cells monolayer from the choriocapillaris. The five layers of Bruch’s membrane consist of the basement membrane of the RPE, the inner collagenous layer, the elastin layer, the outer collagenous layer and the choriocapillaris basement membrane (Fields et al., 2020; Hammadi et al., 2023). The composition and properties of Bruch’s membrane are described in Table 1. The outer retina is avascular and relies on the choroid for nourishment. The choroid is a vascular connective tissue composed of fibroblasts, melanocytes, and immune cells. The innermost structure of the choroid, known as choriocapillaris, resides adjacent to the Bruch’s membrane and plays a crucial role in supporting the outer retina.

RPE cells support the adjacent layer of photoreceptors by regulating the diffusion of nutrients, ions, and metabolic wastes through the oBRB, by phagocyting photoreceptor outer segments, and by absorbing scattering light (thanks to their melanin). This oBRB, therefore, helps maintain the structure and function of the photoreceptors and the choroid. In a diseased state, the oBRB becomes disrupted and the interaction between the choroid and RPE cells becomes impaired, which consequently leads to RPE cells degeneration, photoreceptor cells degeneration, and vision loss (Romero-Vazquez et al., 2021).

### 4.1 Age-related macular degeneration

Age-related macular degeneration (AMD) is a multifactorial disease that progressively causes irreversible central vision loss in the elderly population (≥65 years old) and accounts for about 10% of blindness worldwide (Al-Khersan et al., 2019; Sarkar et al., 2022). AMD can be classified into two forms, dry and wet AMD, and is characterized by the deposition of extracellular deposits, called drusen, between Bruch’s membrane and the RPE (Figure 1), which over time can cause RPE and photoreceptors degeneration in the center of the macula (Thomas et al., 2021). Bruch’s membrane structure and biomechanical properties encounter age-related changes contributing to AMD. Elasticity, permeability, and diffusion reduction are some of these changes that occur due to increased collagen cross-linking, thickness and stiffness, and drusen deposition, respectively (Booij et al., 2010; Tisi et al., 2021).

Dry AMD contributes to 90% of AMD cases, while 10% of patients present wet AMD symptoms. AMD commonly starts with the dry form, then can progress to the wet form (Sarkar et al., 2022). As an impact of choroidal neovascularization, abnormal sub-RPE leaky blood vessels grow from the choriocapillaris into the retina and can lead to the accumulation of fluid or hemorrhage in the retina, which can dramatically reduce vision. (Gryziewicz, 2005; Chakravarthy and Peto, 2020). AMD results from various genetic, environmental and lifestyle risk factors, including aging, oxidative stress, smoking, comorbidity diseases (such as hypertension, hypercholesterolemia, arteriosclerosis, obesity), and single nucleotide polymorphisms (SNPs) in genes like complement factor H and ARMS2/HTRA1 (Lim et al., 2012; Shaw et al., 2016). Oxidative stress is a major contributor of AMD (Jarrett and Boulton, 2012). The RPE resides in a high oxidative stress environment because of their high metabolic activity, photo-oxidative stress from light exposure, presence of photosensitizers like rhodopsin and lipofuscin, and high polyunsaturated fatty acids level. This environment favors ROS production and increases the probability of oxidative damage and cell death of RPE and photoreceptor cells (Hanus et al., 2015; Abokyi et al., 2020; Zhang et al., 2020).

### 4.2 Current treatments and limits

The current gold standard treatment for wet AMD is intravitreal injections of anti-vascular endothelial growth factor (anti-VEGF). This treatment option has proven to slow exudative AMD progression and improve visual outcomes by decreasing retinal fluid and subretinal neovascularization (Hernández-Zimbrón et al., 2018). For dry AMD, nutritional supplements, such as antioxidants and minerals (AREDS and AREDS2 studies) (Age-Related Eye Disease Study Research, 2001; Age-Related Eye Disease Study 2 Research, 2013; Chew et al., 2022), have been reported to prevent the progression from intermediate to more advanced AMD in about 25% of cases. However, despite the higher prevalence of dry AMD, there is currently no effective treatment for reversing dry AMD or for preventing GA (Schmidl et al., 2015). The investigated treatment approaches for dry and wet AMD are summarized in Table 2. This lack of effective treatment options for patients suffering from dry AMD has led to remarkable efforts to identify novel treatment strategies. Tissue engineering of the RPE is one of them.

**TABLE 2:** Proposed treatments for dry and wet AMD.

Mechanism	Drug name	Delivery	Type of AMD	References
ANTI-INFLAMMATORY	• Dexamethasone	Topical application Intravitreal injection Oral administration	Wet AMD	Francis et al., (1996); Ponnusamy et al., (2019); Zhao et al., (2019)
	• Triamcinolone acetonide			
	• Spironolactone			
ANTI-VEGF THERAPY	• Brolucizumab	Intravitreal	Wet AMD	Gragoudas et al., (2004); Spaide Et al., (2006); Falk et al., (2013); Regillo et al., (2022)
	• Bevacizumab			
	• Ranibizumab			
	• Pegaptanib			
	• Aflibercept			
COMPLEMENT INHIBITORS	• Eculizumab	Intravitreal	Dry	Yehoshua et al. (2014)
AAV-BASED GENE THERAPIES	• ADVM-022	Intravitreal and subretinal Suprachoroidal	Dry and wet AMD	Busbee et al., (2021); Khanani et al., (2022)
	• RGX-314			
	• GT-005			
	• HMR59			
NUTRITIONAL SUPPLEMENTS	• Zinc oxide	Oral	Dry AMD	Chew et al., (2013); Bandello et al., (2017); Jacob et al., (2021)
	• Cupric oxide			
	• Lutein			
	• Zeaxanthin			
	• Vitamin C			
	• Vitamin E			
NEUROTROPHIC FACTOR	• CNTF	Encapsulated cell technology	Dry AMD	Zhang et al. (2011)
HYDROXYLAMINE	• OT-551	Topical	Dry AMD	Wong et al. (2010)
VISUAL CYCLE INHIBITORS	• Fenretinide	Oral	Dry and wet AMD	Mata et al. (2013)
STEM CELL TRANSPLANTATION	• hUC-MSCs	Subretinal injection	Dry and wet AMD	Schwartz et al., (2015); Liu et al., (2022b)
	• hESC-RPE			

AMD, age-related macular Degeneration; VEGF, vascular endothelial growth factor; AAV, Adeno-associated virus; CNTF, ciliary neurotrophic factor; hUC-MSCs, human umbilical cord mesenchymal stem cells; hESC-RPE, human embryonic stem-cell derived retinal pigment epithelium.

## 5 Tissue engineering of the retinal pigment epithelium

Tezel and Del Priore described RPE cells as anchorage-dependent cells that should be reattached to a substrate before transplantation to prevent apoptosis (Tezel and Del Priore, 1997). To that end, different biomimetic substrates, natural membranes and synthetic or hybrid scaffolds have been proposed in order to enhance the survival, integration, and differentiation of an engineered RPE monolayer (Rohiwal et al., 2021).

### 5.1 Cell source

ARPE-19 is a human RPE cell line with polarized epithelial cell morphology that expresses genes specific to native RPE cells. ARPE-19 can exhibit functions equivalent to human RPE, like photoreceptor outer segment phagocytosis and epithelial monolayer formation on supportive scaffolds (Dunn et al., 1996; Samuel et al., 2017). Since ARPE-19 cells do not completely resemble human RPE cells transcriptome and functions, do not express certain RPE-related proteins, and might lose some differentiated properties after multiple passages, these cells are valuable only for *in vitro* studies (Samuel et al., 2017; Gupta et al., 2023).

Various cell sources for RPE cell replacement have been suggested. Autologous RPE cells referred to RPE-choroid sheet or RPE cell suspension harvested from the nasal subretinal area of a patient’s eye and manipulated *in vitro* before transplantation is one of them (Assadoullina et al., 2004; Binder et al., 2004; Boulton et al., 2004). Autologous RPE cells may carry the same genetic mutations related to AMD (Binder et al., 2007). Allogeneic fetal RPE cells derived from human fetuses in 15–17 weeks of gestational age have also been used for RPE cell transplantation in the foveal area after surgical removal of the defective RPE layer and the Bruch’s membrane. Immune rejection, however, is the limitation of allogeneic transplantation (Algvere et al., 1994; Algvere et al., 1999).

Pluripotent stem cells like human embryonic stem cells (hESCs) and induced pluripotent stem cells (iPSC) can be differentiated towards an RPE fate using a rapid and directed method described by Foltz and Clegg (Foltz and Clegg, 2017). In brief, stem cells were seeded in plates coated with an ECM-based hydrogel, and cultured for 14 days in the presence of growth factors (insulin growth factors, basic fibroblast growth factor, transforming growth factor beta) and WNT pathway agonist. This protocol generated RPE-like cells that were isolated and cultured in RPE cell maintenance medium for further passage and cryopreservation. The generated RPE cells showed specific polarized, pigmented, and polygonal morphology, expressed RPE markers, and secreted RPE growth factors. This protocol has been used extensively to obtain RPE cells from different stem cell sources (Foltz and Clegg, 2017; Parikh et al., 2022; Regha et al., 2022). Autologous and allogeneic iPSC-derived RPE cells have been employed in human studies. Mandai et al. generated iPSC from dermal fibroblasts of two AMD patients, differentiated them to RPE cells, and transplanted the autologous iPSC-derived RPE cell sheet subretinally (Mandai et al., 2017). In another clinical trial, allogeneic iPS-RPE cells of an HLA homozygous donor were transplanted to 5 HLA-matched patients with wet AMD (Sugita et al., 2020).

Human umbilical cord mesenchymal stem cells (hUCMSC) differentiation to PRE cells using CRX, NR2E1, C-MYC, LHX2, and SIX6 transcription factors is outlined recently by Zhu et al. (2022) hUCMSC-derived iRPE cells were implanted into subretinal space of rat models and demonstrated desirable therapeutic effects. Furthermore, Bone marrow mesenchymal stem cells (BMSCs) treated with ciliary neurotrophic factor were injected intravitreally to diabetic rat eyes. Differentiation of BMSCs to RPE cells was then assessed to evaluate retinal regeneration (Huang et al., 2021).

### 5.2 Biomimetic substrates

Collagen types I, III, IV, and V are major components of Bruch’s membrane, and most of the studies are devoted to develop scaffolds using these ECM proteins. Collagen substrates have been delivered to the subretinal space as crosslinked and non-crosslinked collagen, collagen film, and ultrathin collagen membranes (Susanne, 2011; Murphy et al., 2020). Bhatt et al. (1994) transplanted human fetal RPE cells cultured on type I collagen into the subretinal space of rabbits in two forms: non-crosslinked (soft and flexible) or crosslinked with ultraviolet light (rigid). During the 6 weeks of examination, they observed that RPE cells remained as a monolayer on the non-crosslinked collagen, which complied with the shape of Bruch’s membrane. They suggested that this approach could possibly transfer growth factors or cytokines and prevent retinal degeneration. Other studies investigated RPE cell differentiation characteristics, viability, and morphology after being cultured on thin (10 μm-thick) and ultrathin (7 µm-thick) collagen type I membranes which are approved for clinical use. This easily degradable membrane could be an anchorage for RPE cells to maintain their specific characteristics after subretinal transplantation *in vivo* (Thumann et al., 2006; Thumann et al., 2009). Recently, Yamashita et al. (2023) generated a biodegradable hybrid PCL/collagen nanosheet and grafted it into the subretinal space of a rat model after loading RPE cells on it. This nanosheet showed supportive properties to increase RPE cells survival and monolayer formation with tight junctions. Due to its great mechanical strength, controlled drug delivery, and strong cell adherent property, this RPE cell-loaded PCL/collagen nanosheet could be considered a potent design for cell monolayer transplantation, and retinal degenerative diseases treatment.

Chitosan is a positively charged polysaccharide derived from chitin deacetylation. It is a biocompatible, biodegradable, and mucoadhesive polymer with antibacterial, wound healing, and penetration enhancement properties. Noorani et al. (2018) designed an electrospun gelatin/chitosan nanofibrous substrate that provided a suitable surface for RPE cell adhesion *in vitro.* They disclosed that a mixture of gelatin and chitosan could imitate Bruch’s membrane composition and nanofibrous structure that help RPE cells to preserve their phenotype. Chitosan conjugation with a peptide (serine-threonine-tyrosine) induced tyrosine kinase activity in RPE cells which in turn enhanced photoreceptor outer segment phagocytosis by RPE cells. This conjugated nano chitosan, synthesized by Jayaraman, et al., could therefore serve as a carrier for retinal drug delivery and AMD treatment (Jayaraman et al., 2012).

Silk is mainly produced in the glands of silkworms, commonly *Bombyx mori*, during their metamorphosis. *Bombyx mori* silk has been widely used as a biomaterial for tissue engineering purposes (Kundu et al., 2013; Koh et al., 2015). This material has several advantages including great biocompatibility, chemical modifiability, slow degradation, excellent permeability to water and oxygen, and mechanical strength due to the presence of fibroin proteins (Rockwood et al., 2011). Silk fibroin membranes have been readily exploited as a Bruch’s membrane substitute to support ocular cells attachment, growth, and differentiation (Shadforth et al., 2012). As demonstrated in several studies, RPE cells from different sources were cultured on an ultrathin (mostly 3 µm) and porous *B. mori* silk fibroin membranes to explore RPE cell behavior, adhesion, proliferation, survival, and maturation after cultivation. Considering the results, RPE cell function on a scaffold manufactured from silk fibroin were equivalent to their function on native Bruch’s membrane (Shadforth et al., 2012; Shadforth et al., 2017; Galloway et al., 2018). To optimize the physical properties of silk fibroin membranes, porogens like PEG, crosslinking agents like HRP, polycaprolactone (PCL), gelatin, and elastin (in the form of human recombinant tropoelastin) were incorporated into these scaffolds (Xiang et al., 2014; Shadforth et al., 2015; Suzuki et al., 2019; Belgio et al., 2020). Jeong et al. (2020) produced hydrogels from silk fibroin and mixed it with a synthetic biomaterial, PEG, at different sonication times. RPE cells were isolated from rabbit eyes and seeded on these hydrogels. Scaffold’s porosity, strength, degradation, RPE cell attachment, and proliferation were then measured. Based on the results, the hybrid silk fibroin hydrogel and PEG scaffold prepared with a 20-s sonication period could be a promising material for retinal tissue engineering regarding its beneficial effects on RPE cell growth and gene expression (Jeong et al., 2020).

The self-assembly approach of tissue engineering has been employed to construct tissues without the need for synthetic or exogenous biomaterials (see Section 3.1). Djigo et al. (2019) developed a self-assembled choroidal substitute using choroidal fibroblasts and demonstrated similarities between the engineered tissue and the native tissue concerning ECM composition, thickness, biomechanical properties, and biocompatibility for RPE cells, vascular endothelial cells and choroidal melanocytes. This choroidal stroma, therefore, could become a biomaterial to transplant the RPE reformed on a choroidal substitute.

Other biomimetic biomaterials, such as gelatin (Shakibaie et al., 2018), alginate (Wikström et al., 2008), laminin (Peng et al., 2016), fibrin glue (Ahmed et al., 2015), bioprinted Bruch’s membrane (Kim et al., 2021; Kim et al., 2022), and autologous cryoprecipitate (Farrokh-Siar et al., 1999) have been assessed by several studies as substrates for RPE transplantation into the subretinal space.

### 5.3 Natural membranes

Human amniotic membrane is a thin (20–50 μm), translucid, and elastic tissue that represents the innermost layer of the placenta. Owing to its ECM structural composition (IV and VII collagen, hyaluronic acid, laminin, fibronectin, and proteoglycans), this membrane has exhibited favorable mechanical properties like permeability, cell adhesion, and elasticity (Tosi et al., 2005; Leal-Marin et al., 2021). Additionally, since this tissue can produce different growth factors and cytokines, and is immune-privileged, anti-angiogenic, anti-inflammatory and anti-microbial, various ophthalmic and tissue engineering studies have widely used this natural membrane for decades (Rizzo et al., 2020; Fénelon et al., 2021). Ohno-Matsui et al. (2005) examined RPE cell characteristics cultured on human amniotic membranes and confirmed the preserved epithelial morphology and differentiation phenotype of these cells. Despite its advantages, limitations like low biomechanical consistency and rapid biodegradation have restricted its application (Ma et al., 2010). In this respect, Majidnia et al. (2022) blended human amniotic membranes with a synthetic biomaterial and revealed high porosity, hydrophilicity, cell adhesion, and lack of toxicity attributed to this ultrathin amniotic membrane powder/PCL scaffold generated by electrospinning technique. This hybrid substrate could potentially support RPE cell adhesion, viability, and morphology.

Descemet’s membrane is another natural substrate with features resembling Bruch’s membrane characteristics concerning the abundant presence of type IV collagen, laminin and fibronectin. Daniele et al. (2023) used this membrane to produce a polarized monolayer of human embryonic stem cell-derived retinal pigment epithelial (hESC-RPE) cells *in vitro*.

Discussed biomimetic and natural biomaterials have some drawbacks that make them inefficient for clinical usage and need to be overcome in future studies. Poor biomechanical strength, high biodegradability, difficult processing condition, infection probability, and the possibility of constituents and property changes associated with processing procedures are certain disadvantages related to natural biomaterials. Although some methods like cross-linking have been introduced to enhance their mechanical strength, they have the disadvantage of producing a thicker, non-permeable, and non-biodegradable biomaterial (Karwatowski et al., 1995; Nair et al., 2021; Alvarez Echazú et al., 2022; Wang, 2023).

### 5.4 Synthetic scaffolds

Hydrogels imitate Bruch’s membrane structure and can be physically and chemically cross-linked to natural (collagen, chitosan, alginate, gellan gum, hyaluronic acid) and synthetic polymers (poly (ethylene glycol) (PEG), poly-L-lysine (PLL), poly (3-caprolactone) (PCL), poly (methacrylate-co-meth acrylamide) (PMMA), poly (vinyl alcohol) (PVA), and poly (hydroxyethyl methacrylate) (PHEMA)) to encapsulate cells, stem cells, and drugs (Ballios et al., 2010; Wang and Han, 2017; Park et al., 2018; Cooper and Yang, 2019; Lin et al., 2021; Youn et al., 2022).

Poly (glycolic acid) (PLGA) is an FDA-approved biomaterial with remarkable properties including high biocompatibility, non-toxicity, high cell adhesion, good mechanical properties, and adjustable biodegradation rate. Sharma et al. isolated CD34^+^ cells from peripheral blood of three dry AMD patients to produce oncogenic mutation-free clinical-grade iPSC-RPE patches. They seeded iPSC-RPE cells on PLGA substrates and transplanted them into the subretinal area of immunocompromised rats and pigs. These PLGA iRPE patches significantly improved RPE monolayer integration into the host Bruch’s membrane, differentiation, and functionality (Sharma et al., 2019).

Poly (chloro-para-xylylene) (parylene C) is a FDA approved class IV biocompatible polymer, that is, widely adopted for biomedical applications and drug delivery thanks to its mechanical strength, flexibility, transparency, chemical inertness, conformability, and good encapsulation efficiency (Golda-Cepa et al., 2020; Coelho et al., 2023). In a long-term study, Rajendran Nair et al. (2021b) transplanted human iPSC-RPE cultured on an ultrathin parylene C membrane into the subretinal space of immunodeficient RCS rat models. They assessed iPSC-RPE survival, RPE-specific marker expression, phagocytosis function, and vision restoration after 1, 4, and 11 months. The results revealed survival and functionality of the polarized iPSC-RPE monolayer on this substrate, where RPE survival was observed in 50% of the rats after 11 months. Lu et al. (2012) reduced the thickness of parylene C to the submicron scale (0.15–0.30 μm), which simulated human Bruch’s membrane permeability. The ultrathin (0.30 μm) parylene C was permeable to nutrients and macromolecules needed to nourish RPE cells and could preserve RPE cell features like adherence, proliferation, monolayer formation with tight junctions, and polarization with microvilli. These findings denote that this structure can be an artificial Bruch’s membrane for RPE cell transplantation. Acting as an artificial Bruch’s membrane, the mesh-supported submicron parylene C membrane was used by Koss et al. (2016) to surgically implant polarized human embryonic stem cell-derived RPE (hESCRPE) monolayer in 14 minipig eyes. Retinal preservation, normal RPE and choroid morphology, RPE monolayer survival, lack of local inflammatory response or peri-implant fibrosis were favorable results described in this study.

Polycaprolactone (PCL) is another FDA approved synthetic polymer for controlled intraocular drug delivery. This polymer is identified by its notable slow degradation rate, great *in vivo* biocompatibility, low melting temperature, hydrophobicity, and high porosity nature (Jiang et al., 2020; Waterkotte et al., 2022). Belgio et al. (2021) developed a 3D *in vitro* model of a Bruch’s membrane substitute and an RPE monolayer. They fabricated the Bruch’s membrane substitute using silk fibroin and PCL. The produced Bruch’s membrane showed 44 µm thickness as well as structural and mechanical behavior, biocompatibility, porosity, and permeability comparable to physiological Bruch’s membrane. They concluded that the seeded RPE cells could adhere and grow to this 3D *in vitro* substrate (Belgio et al., 2021). Liu et al. (2022a) employed the electrohydrodynamic jet (EHDJ) printing method to fabricate ultrathin EHDJ-printed PCL scaffolds with small pore sizes for RPE cell culture. This EHDJ-printed PCL substrate was similar to human Bruch’s membrane in terms of biomimetic features such as thickness, biomechanical properties, and permeability, and promoted RPE cells maturation to form a polarized and functional monolayer with tight junctions. Poly (trimethylene carbonate (PTMC) (Sorkio et al., 2017), poly (l-lactic acid) (PLLA) (Thomson et al., 2011), poly (glycerol-sebacate) (PGS) (Neeley et al., 2008), and poly (methyl methacrylate) (PMMA) (Tao et al., 2007) are other FDA approved polymers being used for Bruch’s membrane mimetic substrates with the aim of RPE cells culture and transplantation.

Although synthetic biomaterials are greatly advantageous for ocular tissue engineering applications due to their modifiable properties, reproducibility, good mechanical features, and longer shelf life, there are certain disadvantages associated with these materials, such as lack of cell adhesion on the surface that reflects a need for chemical modification, and immune response or inflammatory reactions induction (Rajendran Nair et al., 2021a; Reddy et al., 2021; Rohiwal et al., 2021).

### 5.5 Functionality assessments of the engineered RPE monolayer

#### 5.5.1 *In vitro* functionality assays

For the RPE, monolayer functionality means measuring barrier integrity, secretion of RPE-specific growth factors, phagocytosis of photoreceptor outer segments, and permeability to macromolecules.

Transepithelial resistance (TER) can be used to measure the RPE monolayer barrier’s integrity, which is related to the correct expression of tight junctions (Subrizi et al., 2012; Kamao et al., 2014). Transmission electronic microscopy can also be used to document the presence of tight junctions, and other structural charateristics of mature RPE, such as microvilli on the apical surface, and melanin granules within the cells (Capeans et al., 2003; Kamao et al., 2014; Shin et al., 2019; Liu et al., 2021b). Immunofluorescent staining is another method to examine the tight-junction marker ZO-1, as well as other specific RPE cell markers like cytokeratin 8 and 18 (intermediate filament proteins), RPE65 (RPE-specific 65 kDa protein), cellular retinaldehyde binding protein (CRALBP), microphthalmia-associated transcription factor (MITF), Bestrophin, and PAX6 (Kamao et al., 2014; Majidnia et al., 2022).

ELISA assay is an approach to assess the concentration of growth factors secreted by RPE cells including pigment epithelium-derived factor (PEDF) and vascular endothelial growth factor (VEGF) (Kamao et al., 2014). The phagocytic activity of RPE cells, which is pivotal for photoreceptor cell maintenance, is tested by phagocytosis assay after incubating RPE cell with fluorescently labeled POS (Zhang et al., 2022).

Proper permeability to nutrients and metabolites is an important feature of the substrates. To assess membranes permeability, the diffusion of Alexa Fluor^®^ 568 Hydrazide sodium salt or Allura Red AC (496.42 Da) across the membrane was measured by calculating the permeability coefficients (Sorkio et al., 2015; Suzuki et al., 2019).

#### 5.5.2 *In vivo* functionality assays (pre-clinical studies)

##### 5.5.2.1 Animal models

Various animal models have been introduced to evaluate the surgical approaches of subretinal implantation of substrates and the biological interactions between the implant and the host retina. To date, rats, rabbits, pigs, and non-human primates have been used for subretinal injections of generated substrates (Koss et al., 2016; Gandhi et al., 2020; Garkal et al., 2022; Rizzolo et al., 2022).

Rats are attractive models on behalf of considerable advantages including easy breeding, ability to be genetically manipulated, cheap maintenance, and quick disease progression. According to limitations of rodent models, these animals have neither macula (which contains rod and cone photoreceptors) nor fovea, do not have drusen deposits similar to humans, and have small size eyes (Koster et al., 2020; Pandi et al., 2021). Royal College of Surgeon (RCS) rat, a FDA accepted model to approve clinical trials, presents RPE degeneration, defective photoreceptor outer segment phagocytosis, and photoreceptor degeneration, that makes it an attractive model for assessing the ability of the engineered tissue to improve or maintain RPE and photoreceptors viability *in vivo* (Fernandez-Robredo et al., 2015; Thomas et al., 2016; Stanzel et al., 2019).

Non-human primates have bigger eye size that facilitates delivery into the back of the eye (Thackaberry et al., 2017). The posterior segment of these eyes has identical structure to human eyes, such as the presence of macula. Drusen deposits with similar composition and location have been reported in Rhesus monkeys (*Macaca mulatta*) and cynomolgus macaque (*Macaca fascicularis*). Limited genetic models, expensive preservation, longer time for AMD induction, and challenging reproduction have restricted their application (Pennesi et al., 2012; Chen et al., 2014; Fletcher et al., 2014).

Pig eyes are the most similar to human eyes between non-primate animals considering their size, retinal structure, and cone distribution (Chen et al., 2014). Yucatán minipigs are example of this model that have been used to assess the feasibility, safety and tolerability of a designed implant (Koss et al., 2016). The breeding and housing on this model is costly, and the intraocular surgery may cause choroidal bleeding and inflammatory responses (Chen et al., 2014).

The retinal structure of rabbits is different from the human eye in the posterior segment. The inner surface of rabbit retina is lined with large vessels that nourishes the retina and there is not a true macula and fovea centralis (Pennesi et al., 2012; Chen et al., 2014; Lossi et al., 2016). In spite of these dissimilarities, rabbit eyes are often used because of the reasonable price for breeding and maintenance and bigger eye size compared to rodents. Rabbit models are being extensively used for scaffolds subretinal transplantation (Chen et al., 2014; Jang et al., 2018; Zhang et al., 2022).

##### 5.5.2.2 Surgical considerations

Immunosuppression and anesthesia are important surgical considerations. For immunosuppression, mouse models have been reported to receive cyclosporin or dexamethasone a day before transplantation until the end of the study. Alternatively, there are immunodeficient mouse models that can be chosen (Little et al., 1996; Stanzel et al., 2019; Rajendran Nair et al., 2021a). Immunosuppression regimens for rabbits include sirolimus, doxycycline, and minocycline administration from 3 days before surgery to the end of experiments and intravitreal triamcinolone administration during the surgery. For pigs, to prevent immune rejection, immunosuppression with rapamycin, tacrolimus, and dexamethasone starts 9 days before transplantation and continues until euthanasia (Koss et al., 2016; Stanzel et al., 2019). Monkeys can receive systemic immunosuppressive drugs like sirolimus, doxycycline, and minocycline from seven to 10 days prior to surgery and throughout the study to eliminate immune rejection (Stanzel et al., 2019; Liu et al., 2021b). General anesthesia with weight-based dosage is usually performed using intraperitoneal injection of ketamine, xylazine, and acepromazine in rodents, intramuscular injection of xylazine into the gluteal muscle of rabbits, inhalation of 2%–5% isofluorane with pigs, and intramuscular injection of ketamine and atropine in non-human primates (Fernandes et al., 2017; Stanzel et al., 2019; Rajendran Nair et al., 2021a; Liu et al., 2021b).

After anesthesia, subretinal graft delivery can be performed by various developed injectors or devices, surgical forceps, and implant instruments. The surgical steps is slightly different in different animals. Generally, the first critical step is to determine the position of the implant. Then the conjunctiva is opened, and the sclera is punctured posterior to the limbus in the temporal superior quadrant. After sclerotomy, paracentesis is performed in the anterior chamber to reduce intraocular pressure to prevent bleeding. In the next step, vitrectomy is performed, the choroid is cut, and a balanced salt solution is injected to create a local retinal bleb. The sheet is then implanted carefully into the subretinal bleb. Fluid-air exchange is done at the end of the operation to reattach the retina. Finally, triamcinolone is injected intravitreally, and dexamethasone/antibiotic ointment is added under lid (Al-Nawaiseh et al., 2016; Stanzel et al., 2019; Gandhi et al., 2020; Rajendran Nair et al., 2021b).

##### 5.5.2.3 Post-operative follow ups

Close post-operative monitoring is necessary to ensure the absence of surgical complications like bleeding, retinal perforation, and misplaced graft. The RCS rat retinas, for instance, are susceptible to acute reactions and surgical trauma because of small eye size (Rajendran Nair et al., 2021b). Ophthalmic imaging devices have helped to monitor the graft with video recordings and multi-color fundus pictures (Liu et al., 2021a). Spectral-domain OCT imaging, fluorescein angiogram, fundus autofluorescence, BluePeak autofluorescence, infrared, and electroretinography, are all *in vivo* retinal imaging methods widely used to follow-up the transplanted engineered tissue after surgery to ensure the proper transplanted membrane position as well as retinal function (Fernandes et al., 2017; Liu et al., 2021a; Zhang et al., 2022). Superior colliculus electrophysiology and optokinetic responses are other recommended approaches to discover visually evoked activities and visual improvement post-transplantation (Rajendran Nair et al., 2021b).

##### 5.5.2.4 Post-mortem analysis

Post-mortem analyses of the transplanted RPE monolayer are mostly oriented towards assessing the presence of the RPE and photoreceptors, scaffold placement and degradation, attachment to Bruch’s membrane, as well as a lack of damage to the surrounding tissues, and the absence of graft rejection. This can be achieved using histology cross-sections and immunostainings.

### 5.6 Human clinical trials

In a prospective, interventional, FDA–cleared phase I/IIa clinical trial (NCT02590692), Kashani et al. (2018) developed a composite subretinal implant, named the California Project to Cure Blindness–Retinal Pigment Epithelium 1 (CPCB-RPE1), that is, being injected to advanced dry AMD patients. This composite is made of a polarized monolayer of hESC-RPE on an ultrathin perylene substrate that mimics the Bruch’s membrane. In 2018, 16 patients had been enrolled in this study. OCT imaging results have shown hESC-RPE and photoreceptor anatomic integration. Implanted eyes showed 17-letter improvement in visual acuity and improved functional activity without vision loss progression. Results support this implant as a potential treatment for patients with severe vision loss from dry AMD (Kashani et al., 2018). In a further study, this group explored in detail the intraoperative surgical procedures and anatomic results (Kashani et al., 2020).

## 7 Conclusion

While many biomaterials have been proposed for the engineering of the corneal endothelium and the RPE using cultured cells, very few have reached the clinical trial phase. Part of the reason resides in the properties of the biomaterial itself. Finding the right combination of thickness, composition, and mechanical properties is a challenge. Part of it is also related to the production of high-quality matured cells that have reformed a functional barrier of high cell density. There is also a need to develop tools to assess tissue quality and *in vitro* functionality. Finally, another challenge is their surgical compatibility, where the engineered tissue must fit the shape and stay in place once implanted, and the surgery itself must not generate complications for the patient. Further research is required to meet these challenges. Tissue engineering holds great promise in generating functional corneal endothelium or RPE deliverable for transplantation in humans.
